# Supplementary material for: Identification of photoperiod-induced specific miRNAs in the adrenal glands of Sunite sheep (Ovis aries)
Source: Front Vet Sci. 2022 Jul 22;9:888207. doi: 10.3389/fvets.2022.888207 (PMC9354845; doi:10.3389/fvets.2022.888207)
Supplement: Supplementary file 1 [file Data_Sheet_1.ZIP › Supplementary file/miRDeep2 file.zip!/miRDeep2 file/Know_Structure/LP42A2/oar-mir-23b.pdf]

miRBase precursor : oar-mir-23b
Total read count : 20851
oar-miR-23b read count : 20816
remaining reads : 35

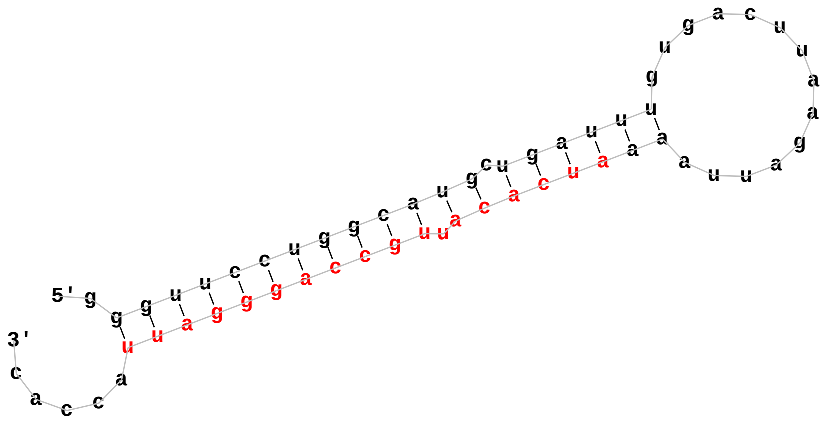

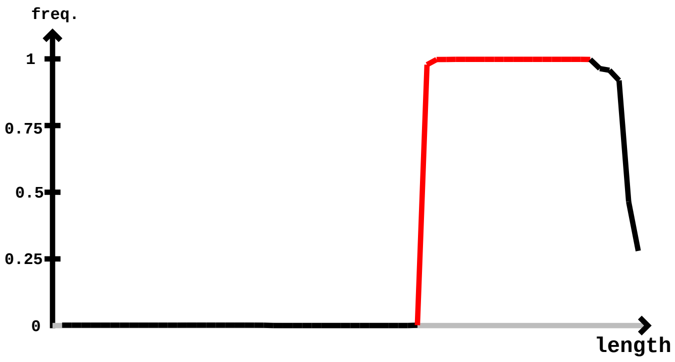


```
                                     oar-miR-23b
5'- ggguuccuggcaugcugauuugugacuuaagauuaaaaucacauugccagggauuaccac   -3'    exp
    ((((((((((((((.((((((..............)))))))).))))))))))..))..    reads      mm          sample
    ggguuccuggcaugcugau.........................................    2          0           seq
    ggguuccuggcaugcugauu........................................    5          0           seq
    ggguuccuggcaugcugauuu.......................................    24         0           seq
    ggguuccuggcaugcugauuug......................................    1          0           seq
    ............ugcugauuugugacuuaagauuaaa.......................    1          0           seq
    ................gauuugugacuuaagauuaaaa......................    1          0           seq
    ....................ugugacuuaagauuaaaa......................    1          0           seq
    ....................................aaucacauugccagggauu.....    6          0           seq
    ....................................aaucacauugccagggauuac...    3          0           seq
    ....................................aaucacauugccagggauuacc..    12         0           seq
    ....................................aaucacauugccagggauuacca.    2          0           seq
    ....................................aaucacauugccagggauuaccac    3          0           seq
    .....................................aucacauugccagggaC......    1          1           seq
    .....................................aucacauugccagggau......    9          0           seq
    .....................................aucacauugcUagggauu.....    2          1           seq
    .....................................aucacauugccagggauu.....    700        0           seq
    .....................................aucacauugccagggCuu.....    2          1           seq
    .....................................aucacauugcUagggauua....    1          1           seq
    .....................................aucacauugccagggauua....    127        0           seq
    .....................................aucacauugccagggauuac...    779        0           seq
    .....................................aucacauugccagggauuacc..    9331       0           seq
    .....................................aucacauugccagggauuacca.    3765       0           seq
    .....................................aucacauugccagggauuaccac    5652       0           seq
    ......................................ucacauugccagggauu.....    2          0           seq
    ......................................ucacauugcUagggauu.....    1          1           seq
    ......................................ucacauugccagggauua....    3          0           seq
    ......................................ucacauugccagggauuac...    13         0           seq
    ......................................ucacauugccagggauuacc..    131        0           seq
    ......................................ucacauugccagggauuacca.    77         0           seq
    ......................................ucacauugccagggauuaccac    178        0           seq
    .......................................cacauugccagggauuacc..    2          0           seq
    .......................................cacauugccagggauuacca.    2          0           seq
    .......................................cacauugccagggauuaccac    1          0           seq
    ........................................acauugccagggauuacc..    6          0           seq
    ........................................acauugccagggauuacca.    1          0           seq
```

oar-miR-23b

```
ggguuccuggcaugcugauuugugacuuaagauuaaaaucacauugccagggauuaccac
........................................acauugccagggauuaccac    4    0    seq
```
